# On Puerperal Fever

**Published:** 1804-07-01

**Authors:** 


### *To the Editors of the Medical and Physical Journal.*

GENTLEMEN,

IT has frequently occurred to me, that your excellent Journal may be still more enriched with useful information, and that without any deviation from the plan you have so successfully adopted. It is but too well known to practitioners, that there are some diseases which, though not considered incurable, are yet extremely fatal, and from the rare instances of recovery, it may be inferred that neither their nature, nor proper mode of treatment, has yet been ascertained; at least, it would be impossible to shew shew that further advancement towards a cure might not be discovered. To collect into one point of view the experience and observations of those whose situations have given them frequent opportunities of attending to such diseases, would be a most desirable object.

No publication is better adapted for the conveyance of such communication to the public than the Medical Journal.

If this proposition meets your approbation, and that of your numerous correspondents, I would take the liberty of proposing, as the first subject of enquiry, Puerperal Fever, a disease fatal in the extreme, and not till lately much attended to. For myself, I can only say, that I have had frequent opportunities of observing it, and have known all the remedies which have been recommended, tried, but without success. Some of them even appeared to hasten the fatal catastrophe.

A case lately came under my care in which the alkalies, so much boasted of on the Continent, had a complete trial, without producing any change on the disease, and the patient died the ninth day.

I am, &c.

*May* 25, 1804. H.

